# The 6-min walk test as a new outcome measure in Amyotrophic lateral sclerosis

**DOI:** 10.1038/s41598-020-72578-3

**Published:** 2020-09-23

**Authors:** Massimo Russo, Christian Lunetta, Riccardo Zuccarino, Gian L. Vita, Maria Sframeli, Andrea Lizio, Stefania La Foresta, Cristina Faraone, Valeria A. Sansone, Giuseppe Vita, Sonia Messina

**Affiliations:** 1 Unit of Neurology and Neuromuscular Disorders, Department of Clinical and Experimental Medicine, University of Messina, AOU Policlinico “G. Martino”, 98125 Messina, Italy; 2 NEuroMuscular Omnicentre of Milan, Milan, Italy; 3 NEuroMuscular Omnicentre of Arenzano, Arenzano, Italy; 4 NEuroMuscular Omnicentre of Messina, University Hospital “G. Martino”, Messina, Italy; 5 Neurorehabilitation Unit, University of Milan, Milan, Italy

**Keywords:** Neuroscience, Diseases of the nervous system, Amyotrophic lateral sclerosis

## Abstract

One of the issues highlighted in amyotrophic lateral sclerosis (ALS) clinical trials is the lack of appropriate outcome measures. The aim of this multicentric study was to evaluate the 6-min walk test (6MWT) as tool to monitor the natural history of a cohort of ALS patients followed up over a 6-month interval. Forty-four ambulant patients were assessed at baseline and after 1, 3 and 6 months. Eight out of forty-four lost the ability to walk before the end of the study. The 6MWT and the objective measures linked to motor function, such as 10 m walking test (10MWT) and Time-up and go (TUG), the ALSFRS-R and the ALSFRS-R items 7–9 showed a good responsiveness to change over the 6-month interval. There was a strong correlation between 6 and 10MWT, TUG, ALSFRS-R, ALSFRS-R items 7–9 and FVC% at baseline. There was no correlation with Edinburgh Cognitive and Behavioural ALS Screen (ECAS) and Modified Borg Scale (MBS). The Δ of 6MWT from T0 to T6 significantly correlated with the Δs of 10MWT and TUG. There was no correlation with the Δs of ALSFRS-R, ALSFRS-R items 7 9, ECAS, MBS and FVC%. The discordance between changes of the 6MWT and ALSFRS-R at 6 month highlights the different content validity among these instruments. The concordance among 6MWT, 10MWT and TUG indicates that the 6MWT is an objective, sensitive and robust tool to measure motor performances in a longitudinal setting. The main limitations of our study were the small sample size and the high percentage of patients (18%) lost at follow-up. Therefore, further studies on larger cohorts, and exploring the relation between 6MWT and need of ventilator support or survival could strengthen our results.

## Introduction

Amyotrophic lateral sclerosis (ALS) is a neurodegenerative disease of the human motor system. Survival in ALS is dependent on several factors, including clinical presentation, rate of disease progression, early presence of respiratory failure and the nutritional status of patients^1^. The Functional Rating Scale (ALSFRS) and more recently the ALSFRS-Revised (ALSFRS-R) are used to measure functional decline and to evaluate treatment effects on function in clinical trials as primary endpoint. However, the ALSFRS-R is a subjective tool as is a patient-reported outcome measure (OM). Furthermore, it explores area of impairment such as speech or salivation that may not ameliorate in therapeutic trials diluting the responsiveness of this tool to treatments. Moreover, there is no consensus upon the threshold at which a change in ALSFRS-R score leads to an important transition in functional status^2^. Adequate daily activity, specifically walking, has been demonstrated to be strongly linked to life expectancy, morbidity and quality of life (QoL) in other neurological conditions, such as stroke^3^. Furthermore, walking capacity has been shown to be the only independent predictor of survival when compared to other functions of activity of daily living in ALS patients^4,5^.

The 6-min walk test (6MWT) evaluates the global and integrated responses of all the systems involved in walking, including the pulmonary and cardiovascular systems and neuromuscular units. It has been applied widely for evaluation of functional exercise capacity in neurological diseases such as stroke, Parkinson’s disease, cerebral palsy, multiple sclerosis fibromyalgia, and spinal cord injury^6–11^. The 6MWT has been demonstrated to be a reliable OM in several neuromuscular disorders such as, myotonic dystrophy^12^, spinal and bulbar muscular atrophy^13^, chronic spinal poliomyelitis^14^, Pompe’s disease^15^, diabetic neuropathies^16^, Duchenne muscular dystrophy^17^ and in chronic inflammatory polyneuropathy^18^. In a multicentre prospective study in adult patients with Charcot–Marie–Tooth disease, we have already demonstrated the validity and reliability of the 6MWT with a good correlation with other OM commonly adopted in this disease^19,20^.

Recently, in a cross sectional study in a cohort of ALS ambulant patients, the 6MWT resulted a valid measure of walking capacity correlated to lower extremity muscle strength and independent from ALSFRS-R and forced vital capacity (FVC). Authors of this study proposed 6MWT as a quantitative, simple, and inexpensive OM of walking capacity for early stage clinical trials in ambulant patients with ALS^21^. On the contrary, no longitudinal studies have been performed to test the reliability and sensitivity to change of the 6MWT in ALS. This information appears to be very valuable, since a better understanding of the possible functional changes over time in untreated patients is essential when planning clinical trials.

The aim of this study was to report a multicentre effort to perform a natural history study including a large cohort of ALS patients followed up longitudinally with the use of the 6MWT. More specifically, we were interested in establishing the correlations among 6MWT, patient-reported OMs, such as the ALSFRS-R, and other functional measures commonly used in ALS over a 6-month interval. We also aimed to evaluate the impact of cognitive and respiratory impairment.

## Results

**Figure 1:**
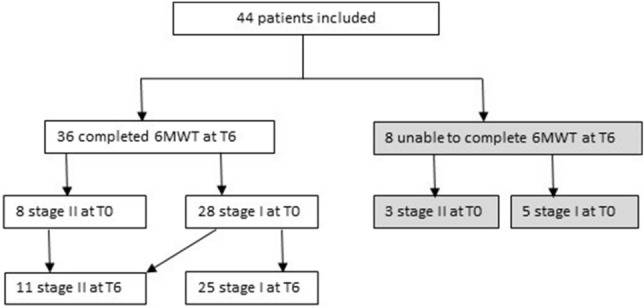
Patient’s stage.

Forty-four ALS patients were included in the study (Table 1). Eight out of forty-four patients have lost the ability to walk during the 6-month period of the study. In detail, one of these have lost the ability to walk after the baseline evaluation, while the others 7 patients between the T3 and the T6 timepoints. At T0 three out of eight were in stage II: one patient was able to walk with sticks, one on crutches and the other with stick plus right ankle foot orthosis. Comparing the baseline demographic and clinical features of patients able to complete the 6MWT at T6 with those of the drop-out patients, the drop-out cohort reported a significantly lower ALSFRS-R (34.12 ± 3.27 vs 37.47 ± 5.53, *p* = 0.0167) and a shorter 6MWT, although not statistically significant (285.06 ± 133.35 vs 369.89 ± 115.18, *p* = 0.1206). Thirty-six patients were able to complete the 6MWT at T6. At T0, 28 were in stage I, while 8/36 patients were in stage II. At T6, 3 patients progressed from stage I to stage II (1 ankle foot orthosis, 1 walker, 1 ankle foot orthosis plus walker) (Fig. 1).

**Table 1:** Participants clinical characteristics.

Gender (male/female)	30/14
Age (years)	60.9 ± 9.25 (33.8–80)
Disease duration (months)	17.2 ± 6.19 (4–24)
Site of onset (spinal/bulbar)	30/14
ALS stage at T0: I/II	33/11
ALS stage at T6: I/II/III	25/11/8
ALSFRS-R at T0	36.9 ± 5.26 (20–45)

Mean ± SD (range).

The 44 patients able to complete of the test–retest evaluations for the 6MWT, Time up and go (TUG) and 10MWT demonstrated excellent reliability with an Intraclass Correlation Coefficient (ICC) value of 0.99 [0.98–0.99], 0.92 [0.86–0.96] and 0.98 [0.96–0.99], respectively.

Considering the longitudinal analyses, a multiple imputation for longitudinal data was assessed to address the missing data, considering the 18% of missingness for each variable as a statistical issue in the power of the analysis considering our small sample size. Statistical analysis showed a significant worsening of clinical function over time, reporting a statistically significant differences from baseline values to both T3 and T6 in the 6MWT, 10MWT and Time Up and Go (TUG) (Table 2). Conversely, at T1, the above mentioned OMs did not show statistically significant changes from baseline values. As in the above-mentioned OMs, also the patient-reported clinical status evaluated by ALSFRS-R and ALSFRS-R ITEM 7–9 showed a significant decline already evident at T3 and confirmed at T6 (Table 2).

**Table 2:** Responsiveness of 6MWT, 10MWT, TUG, ALSFRS-R and ALSFRS-R item 7–9.

	T0	T1	T3	T6
6MWT	354.47 ± 120.35*^§^	352.71 ± 129.70	324.38 ± 135.62*	297.01 ± 133.86^§^
Adjusted ES		0.01	0.22	**0.56**
10MWT	10.68 ± 5.15*^§^	10.95 ± 4.80	12.45 ± 5.84*	14.27 ± 7.08^§^
Adjusted ES		0.03	0.19	**0.59**
TUG	11.15 ± 5.46*^§^	11.13 ± 4.76	12.76 ± 6.71*	14.13 ± 6.61^§^
Adjusted ES		< 0.01	0.36	**0.53**
ALSFRS-R (total)	36.86 ± 5.32*^§^	35.09 ± 5.93	32.55 ± 6.72*	29.89 ± 7.38^§^
Adjusted ES		0.32	**0.71**	**0.98**
ALSFRS-R (7–9)	9.18 ± 2.30*^§^	8.55 ± 2.44	7.66 ± 2.42*	6.89 ± 2.73^§^
Adjusted ES		0.27	**0.62**	**0.92**

Mean ± SD (range).

*^,§^Significant differences at *p* < 0.01.

The adjusted ES threshold levels were defined as follows: ‘trivial’ (< 0.20), ‘small’ (≥ 0.20 < 0.50), ‘moderate’ (≥ 0.50 < 0.80), or ‘large’ (≥ 0.80).

In bold adjusted ES with moderate or large threshold.

Among the objective OMs, 6MWT, 10MWT and TUG all showed a “moderate” responsiveness to change at T6 (aES = 0.56 for the 6MWT, 0.59 for the 10MWT and 0.53 for the TUG) (Table 2).

ALSFRS-R and ALSFRS-R IT 7–9 showed a ‘large’ responsiveness at T6 that was 0.98 for the ALSFRS-R and 0.92 for the ALSFRS-R IT 7–9, and a “moderate” responsiveness at T3 that was 0.71 and 0.72 respectively (Table 2).

**Figure 2:**
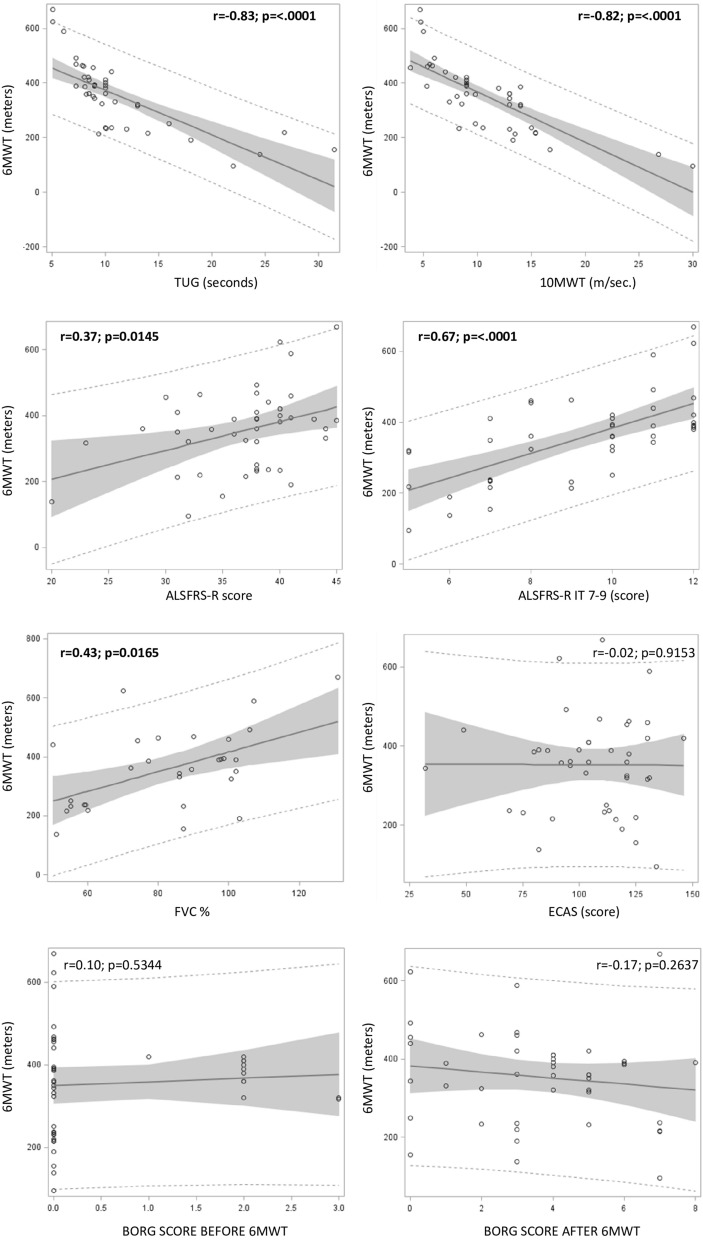
Correlation between 6MWT and other OMs at T0. Significant values are in bold.

**Figure 3:**
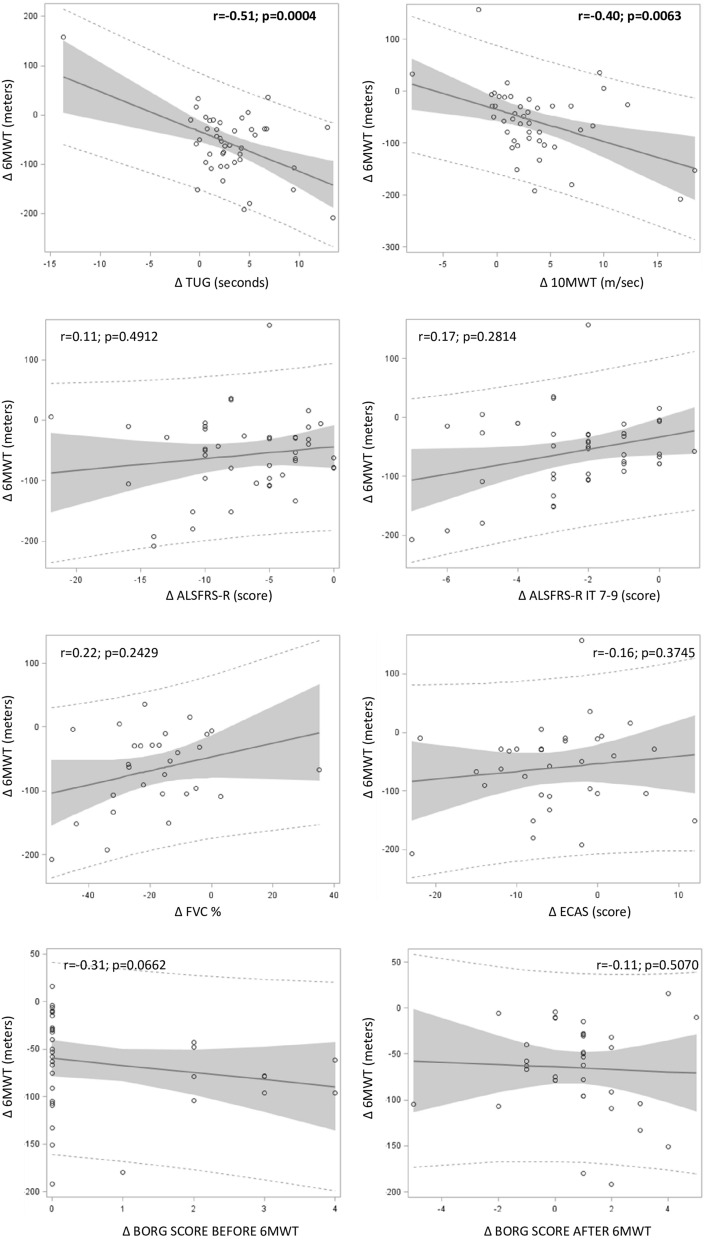
Correlation between 6MWT Δ and other OMs Δ from T0 toT6. Significant values are in bold.

There was a strong correlation between 6 and 10MWT, TUG, ALSFRS-R, ALSFRS-R items 7–9 and FVC% at baseline. There was no correlation with Edinburgh Cognitive and Behavioural ALS Screen (ECAS) and Modified Borg Scale (MBS) (Fig. 2). The Δ of 6MWT from T0 to T6 significantly correlated with the Δs of 10MWT and TUG. There was no correlation with the Δs of ALSFRS-R, ALSFRS-R items 7 9, ECAS, MBS and FVC% (Fig. 3).

## Discussion

One of the issues highlighted in ALS clinical trials is the lack of appropriate OMs. The most used OMs include FVC, hand-held dynamometry (HHD), ALSFRS-R and survival. However, each of them has some benefits and some critical points. It is easy to evaluate FVC and it has a good correlation with survival, but on the other hand site of onset affects timing of FVC decline and muscular weakness of orbicularis oris muscle may hind the performance^22^.

HHD is clinically relevant and very objective, but requires rigorous training, relies upon examiner strength and patient effort. Indeed, it depends on the strength of the evaluator that may overpower the patient’s strength, therefore resulting unreliable when testing strong muscles. To detect very accurately and reliably patient’s strength, an innovative device has been proposed, able to catch smaller changes in strength, and consequently with a higher sensibility compared to ALSFRS-R or FVC^23^. Unfortunately, the device is still not broadly available.

ALSFRS-R is reproducible and easily administered but at the same time is subjective and affected by symptomatic treatment (e.g. anticholinergic drugs for sialorrhea)^23^. Furthermore, about a seventh of patients with ALS of a pooled clinical trial database (PRO-ACT) in the placebo group has been shown a plateau or an improvement in ALSFRS-R score over a 6-month period^24^. This finding does not match the routine clinical experience, the biologic reality of ALS disease course nor the results on objective OMs^25^.

Finally, survival evaluates the true effect of the therapeutic intervention, but increases trial duration and costs and does not provide information about disability or QoL^22^. Recently, to complement the ALS outpatient clinic visit and to enable remote monitoring of disease progression, authors have proposed patients reported OMs as useful tool that correlate with other validated ALS OMs^26,27^.

The 6MWT has already showed a good correlation with the other OM currently used in ALS, and among these is the one that more closely reflects the ability to walk in real life^21^. Our results confirm the previously reported strong correlation between 6MWT and TUG, 10MWT and ALSFRS-R^21^. We have also found a good correlation with FVC, suggesting that the pulmonary function may have a crucial role in determining the 6MWT result even in ALS patients. Differently from Sanjak el al.^21^ we found a good correlation with FVC even in stage II patients, although in stage I it is stronger. The lack of correlation between 6MWT with MBS and ECAS highlights that neither the perception of the symptoms nor the cognitive abilities significantly influence the results when motor and respiratory deficits are present.

Our data show for the first time the 6MWT performance in a longitudinal setting. All the OMs showed a significant decline at T3, even more evident at T6. At T6 we found a similar responsiveness for 6MWT, TUG and 10MWT. In our cohort, the ALSFRS-R and ALSFRS-R 7–9 showed the highest responsiveness to change after 3 and 6 months, confirming its valuable role in assessing the ALS multidisciplinary decline. However, when we consider the correlation among the Δs of the different OMs, the 6MWT correlated only with the TUG and the 10MWT. The discordance between changes of 6MWT and ALSFRS-R at 6 month highlights the different content validity among these instruments. The ALSFRS-R covers various aspects of the disease, such as difficulties in speech, swallowing, handwriting, cutting food, presence of excessive salivation or dyspnea etc. and only an item focuses on walking. Therefore, this discordance of changes over a 6-month period is what we see in our clinical practice, where the ALS phenotypes can present differently and can evolve with a different speed of deterioration for each apparatus. However, the main limitation of the use of ALSFRS-R in clinical trials is the subjectivity of the measure and that some of the items are not able to assess a possible amelioration in motor function, which is a crucial point for ALS patients. On the other hand, 6MWT might demonstrate a rapid curve of decline in ambulatory capacity and this can be a limitation to test new compounds with relatively mild efficacy. These new findings underline the possible additional value of using a combination of OMs including the 6MWT in the stratification process and in the design of clinical trials.

## Conclusions

In conclusion, considering the wide use of 6MWT in other neuromuscular diseases, its concordance with other OMs used in ALS and the good responsiveness and reliability in a longitudinal setting, the present results support 6MWT as a quantitative, objective, simple-to-use and inexpensive OM of walking capacity for early stage clinical trials in ambulatory ALS. We propose a multifaceted approach including objective and patient-reported tools, to better highlight the effects of upcoming new therapeutic approaches. The main limitations of our study were the small sample size and the high percentage of patients (18%) lost at follow-up, therefore further studies on larger cohorts and exploring the relation between 6MWT and need of ventilator support or survival could strengthen our results.

## Subjects and methods

This is a prospective longitudinal multicentric cohort study involving ALS patients recruited from 3 tertiary neuromuscular centres and followed for 6 months. A specific training of physicians, psychologists and therapists from the three neuromuscular centres in the application of the study instruments was performed.

Inclusion criteria were:

1. Clinically probable, laboratory supported probable or clinically definite diagnosis of ALS according to the modified El Escorial criteria.
2. Age in the range 25–80 years.
3. Ability to walk at least 75 m at 6MWT without or with the aid of assistive devices (walker, cane or ankle–foot orthoses).
4. Disease duration < 24 months.
5. FVC > 50%.
6. Patients able to understand, to comply with the requirements of the entire study, including the caregiver compliance, and to give an informed consent.

Exclusion criteria were:

1. A cognitive impairment interfering, according to the principal investigator of the centre, with the capacity to fully understand the informed consent and to perform the study procedures.
2. Use of non-invasive ventilation.
3. Percutaneous oxygen saturation (SpO_2_) < 90% using pulse oximetry at the finger during the 6MWT.
4. Presence of other diseases that may severely affect walking ability.
5. Use of experimental drug or participation in a clinical trial within 3 months prior to screening.

Patients were assessed with the 6MWT at baseline (T0), after 5 ± 2 days for retest and after 1 (T1), 3 (T3) and 6 months (T6). For ratings of perceived exertion before and after the 6MWT, the MBS was administered to patients. At the same time points, 10 m walking test (10MWT), TUG, ALSFRS-R were also performed. FVC was evaluated at T0 and T6. Cognitive involvement was explored with ECAS at T0 and T6.

We subdivided patients according to their ambulatory status in three groups: patients ambulatory without the use of an assistive device were considered in stage I, patients ambulatory with the aid of an assistive device were defined as stage II, non-ambulant patients were definite as stage III^21^.

The study was approved by the “Ethical committee of Messina, University of Messina”. All methods were performed in accordance with the relevant guidelines and regulations. Written informed consent was obtained from all the participants.

### 6MWT

The 6MWT was performed according to the American Thoracic Society guidelines^28^. Participants walked on a flat hard surface wearing shoes, unassisted or using their usual device, 25-m track marked by 2 cones. Participants were instructed to walk as safe and as far as they could for 6 min. For patients’ safety, SpO_2_ was recorded during the completion of the 6MWT using fingertip pulse oximeter. The test has been widely used in other neuromuscular disorders such as Duchenne muscular dystrophy and our group previously showed significant correlations of the 6MWT and other functional tests and health-related QoL tools^29–31^.

### MBS

The MBS is and easy visuo-analogic scale that provides rapid information about a patient’s subjective state of dyspnoea. It is rated from 0 to 10, being 0 equal to “no breathless at all” while 10 equal to “Maximal”^32^.

### 10MWT

The 10MWT assesses functional mobility and walking speed in meters per second over a short duration. It sets a total distance of 14 m, marked at the beginning and end 2 m with tape, and the subject makes a 14-m distance from the sign “start”. Time is measured except beginning and end 2 m for acceleration and deceleration. A lower value indicates a slower gait speed^33^.

### TUG

The TUG was performed to assess test lower extremity function, mobility and fall risk. The test uses the time the patient takes to rise from a standard-height chair with arm at rest, walk 3 m, turn around, walk back to the chair and sit down and it was recorded using a handheld stopwatch^34^. Stage II participant performed the test with their assistive device.

### ALSFRS-R

The ALSFRS-R is a patient-reported outcome. It consists of an ordinal rating scale questionnaire that encompasses four domains of function: bulbar, gross motor, fine motor, and respiratory functions. In total it focuses on 12 functional activities relevant in ALS, with a rate between 0 (loss of function) and 4 (normal function)^35^. The scale has shown high internal consistency, test–retest reliability and sensitivity to functional change and is the most commonly used tool to monitor functional changes in ALS clinical trials. We also considered as separated subscore the items 7–9 focusing on walking, climbing stair, and turning in bed items, and therefore more related to ambulation.

### FVC

FVC was evaluated using a portable handheld spirometer following standard procedure^36^. Participants performed 3 trials and the best result was considered and reported as percent predicted compared to age, height, and gender norms using reference values included in the spirometer.

### ECAS

Patients’ cognitive and behavioural disability was assessed by the two ECAS specific screens. The ECAS-cognitive screen consists of 16 items organized into two sub-scales. An ALS-specific sub-scale focuses on the cognitive domains of language, verbal fluency and executive and social functions. A non-ALS-specific sub-scale assesses memory and visuospatial function. The sub-scales of the ECAS-cognitive screen range, respectively, from 0 to 100 and from 0 to 36. Low scores indicate a greater deficit. The ECAS-behavioral screen is an interview of the carer. It includes assessment of five domains of behaviour of ALS patients. The sub-score for behavioral change ranges from 0 to 10. High scores indicate a more severe involvement^37^. The ECAS feasibility in ALS has been confirmed administering longitudinally this test in comparison with standard neuropsychological tests^38^.

### Statistical analysis

Continuous variables are presented as mean ± standard deviation (SD) and categorical variables as frequencies and percentages.

Statistical analysis was performed by GraphPad Prism, version 7.00 (GraphPad Software, La Jolla, CA, USA) and SAS/STAT software version 9.3 (SAS Institute Inc., Cary, NC). The relation between the 6MWT and other functional data, both at baseline and considering their changes over time, was assessed using the non-parametric Spearman’s correlation coefficient.

To compare the cohort of patients that were able to performed the 6MWT at T6 with the cohort of drop-out patients, the non-parametric Mann–Whitney U test for continuous variables and the chi-square test for the dichotomous ones were used.

6MWT reproducibility was assessed using the Intraclass Correlation Coefficient (ICC).

Considering the longitudinal analysis, in case of missing data, a multiple imputation (MI) for longitudinal data was used to simulate missing values and have full availability of data for each considered variable. A mixed-effects model for repeated measures was used to estimate a 6-months change in the 6MWT and in the other OMs. Responsiveness of 6MWT and other OMs was assessed by calculating the standardized response mean (SRM) as the mean baseline to 1, 3 and 6 months change in score divided by the SD of the individual’s score change. To avoid over or underestimation of magnitude of the change over time, adjusted effect size (aES) was calculated with the formula: SRM ∗ √2 ∗ √(1 − r), where r = Spearman correlation. The aES threshold levels were defined as follows: ‘trivial’ (< 0.20), ‘small’ (≥ 0.20 < 0.50), ‘moderate’ (≥ 0.50 < 0.80), or ‘large’ (≥ 0.80)^39^. All hypothesis tests conducted were 2-tailed. A *p* value < 0.05 was considered statistically significant.
